# Efficacy of transformational breath for anxiety management in professional voice users

**DOI:** 10.1371/journal.pmen.0000119

**Published:** 2025-06-20

**Authors:** Philippa Charlotte Rose Wheble, Dehan Elcin

**Affiliations:** 1 Performing Arts Medicine, Division of Surgery and Interventional Sciences, University College London, London, United Kingdom; 2 Department of Psychology, Tulane University, New Orleans, Louisiana, United States of America; David Geffen School of Medicine: University of California Los Angeles David Geffen School of Medicine, UNITED STATES OF AMERICA

## Abstract

Music Performance Anxiety, a subset of social anxiety disorder (SAD), can significantly impede the lives of professional voice users (PVUs). This pilot cohort study evaluates the efficacy of Transformational Breath (TBr), a facilitated conscious breathing technique that employs high ventilation breathing, as a therapeutic intervention for anxiety management in PVUs. We recruited PVUs diagnosed with mild to moderate SAD and randomly assigned them to an intervention group (n = 12) that completed three TBr sessions with a certified practitioner, or to a waitlist control group (n = 12). Both groups were assessed using a battery of psychological (Generalised Anxiety Disorder Scale (GAD-7), Patient Health Questionnaire (PHQ-9), Social Phobia Index (SPIN), Kenny’s Music Performance Anxiety Inventory (K-MPAI), Warwick-Edinburgh Mental Well-Being Scale (WEMWBS)) and physiological (blood pressure, heart rate, respiratory rate, oxygen saturations, peak expiratory flow rate) measures on three separate occasions. The outcome measures were evaluated before and after each session in the intervention group. No Group x Time interaction was found for any of the physiological or psychological measures. However, a significant Time main effect was found for K-MPAI (X2(5)=20.157, p = .001) and GAD-7 (X2(5)=12.79, p = .025) within the TBr group. Post hoc Wilcoxon-signed rank tests with Bonferroni correction for multiple comparisons revealed significant differences between K-MPAI scores at time points Post 3 and Pre 3 (z = 3.00, adj. p = .040), Post 3 and Pre 2 (z = 3.27, adj. p = .016) and Post 3 and Pre 1 (z = 2.95, adj. p = .048). No pairwise differences were found for GAD-7. The pattern of results suggests that TBr may be effective in acutely decreasing music performance anxiety.

## Introduction

Professional voice users (PVUs) frequently experience Music Performance Anxiety (MPA), a specialized form of social anxiety disorder (SAD) that can precipitate symptoms ranging from mild nervousness to debilitating panic attacks in a performance setting [1]. MPA has a high prevalence of 59% among musicians, with 21% reporting this as severe [2]. Its reach extends beyond musical performance, influencing broader social and professional interactions in addition to mental health. This condition often coexists with other anxiety disorders and depression, amplifying its impact on personal and professional well-being [3].

Hyperventilation and dysfunctional breathing in response to perceived danger cause hypocapnia, which drives the physiological, behavioural and cognitive cycle of anxiety [4–8]. Breathing modification can profoundly influence respiratory physiology and neurophysiology to regulate behaviour, cognition, and emotion [6,9], relieve anxiety and improve physical health [7]. MPA is strongly correlated with hyperventilation in the pre-performance setting [10], suggesting that conscious breathing practices may address dysfunctional breathing and anxiety in PVUs.

### Breathwork and anxiety

Breathwork “comprises various practices which regulate the way that one breathes, in order to promote mental, emotional & physical health” [11] and is becoming a popular intervention to modify conditioned breathing patterns that are implicated in anxiety responses. There are hundreds of practices, each with distinct philosophies and breathing patterns. It is important to differentiate between practices to explore how breathing mechanics may exert different influences on physiology, mental health, and wellbeing.

With some risk of oversimplification, breathwork techniques can be divided into two main categories: slower-paced breathwork techniques, defined as less than 10 breaths per minute [12,13] and faster-paced breathwork techniques described as high ventilation breathwork (HVB) [14]. We define HVB as voluntarily increasing the tidal volume and breathing rate to exceed a normal respiratory rate of 12–20 breaths per minute.

Transformational Breath (TBr) is a HVB practice that uses fast-paced, diaphragmatic breathing without relying on accessory muscles. In a typical 60-minute session, certified practitioners guide individuals to maintain a connected breath pattern, with an uninterrupted flow and a relaxed, complete exhalation. While rate and tidal volume are important, the inspiratory/expiratory ratio has also been shown to modulate the autonomic nervous system and moderate the stress response. Prolonging the exhale appears to promote parasympathetic nervous system activation and relaxation [15]. TBr maintains the inhale at approximately twice the duration of the exhale and likely promotes sympathetic nervous system (SNS) arousal during the practice. HVB has been shown to acutely increase SNS activity characterized by elevated heart rate, increased blood pressure, and enhanced electrodermal activity [14], but the longer-term physiological and psychological effects are unknown. Two small studies have demonstrated a reduction in anxiety symptoms using similar HVB practices to TBr [16,17].

Cognitive Behavioural Therapy (CBT), the gold standard treatment for anxiety [18], commonly uses brief hyperventilation interventions [19]. These fast, shallow breathing exercises recreate the hypocapnia, internal sensations and physical experiences of anxiety in a process called interoceptive exposure (IE). Therapeutic IE acclimates individuals to the physiological sensations of anxiety, reduces sensitivity and modulates individual responses to SNS activation [20]. Prolonged and intense IE with coaching to promote mindful observation, acceptance, re-interpretation, and response to the experience of arousal has been shown to improve CBT outcomes [7,21–23]. A typical TBr session includes 45 minutes of HVB, delivering prolonged therapeutic interoceptive exposure, and integrates various techniques to support the subject through the experience in a similar methodology of mindful acceptance to CBT. The practitioner offers feedback on the subject’s breathing, therapeutic touch and positive affirmations throughout the practice, and strategies including toning, movement and breath-holding are employed, which we suspect modulate the effects of hypocapnia during the prolonged practice.

Evidence suggests that PVUs display high levels of interoceptive sensitivity [24], making TBr and HVB an important area of research for anxiety management in this group. This study aims to investigate the effectiveness of TBr in the management of performance anxiety in PVUs.

## Methods

### Ethics statement

This research was approved by the University College London Research Ethics Committee with approval number 8119/001. Formal written consent was obtained from all participants prior to their participation in the study.

### Enrolment, eligibility and randomization

Twenty-four participants were recruited via email from institutions including the British Association for Performing Arts Medicine, Royal College of Music, Trinity Laban Conservatoire, Guildhall School of Music, and Royal Academy of Music between 10^th^ March and 10^th^ April 2016. Eligible participants were professional singers or singers in professional training who exhibited a Social Phobia Inventory (SPIN) score of higher than nineteen, indicating current mild-moderate social anxiety, who had no prior experience with TBr. Individuals with severe depression or anxiety, current suicidal ideation, or those who smoked, consumed alcohol more than weekly recommended limits, regularly used beta-blockers, anti-hypertensive medication, or inhaled asthma medication were excluded. Volunteers meeting the inclusion criteria (n = 24) were randomly assigned to the intervention (n = 12) or control group (n = 12) by PW in the order that they registered for the study.

### Outcome measures

#### Psychological assessments.

- Social Phobia Inventory (SPIN): A 17-item questionnaire assessing the severity of Social Anxiety Disorder (SAD) symptoms, each rated from 0 (none) to 4 (extreme). The total score ranges from 0 to 68, with scores above 19 indicating a diagnosis of SAD. This test demonstrates good test-retest reliability and high correlation with other measures of anxiety [25].
- Generalized Anxiety Disorder Scale (GAD-7): A 7-item questionnaire assessing the frequency of anxiety symptoms, each rated from 0 (none) to 3 (every day). The total score ranges from 0 to 21, with scores above 10 suggesting a significant level of anxiety [26].
- Patient Health Questionnaire (PHQ-9): A 9-item questionnaire assessing the frequency of depression symptoms, rated from 0 (none) to 3 (every day). The total score ranges from 0 to 27, with scores above 10 indicating a presence of moderate depressive symptoms [27]. The test is characterized by 88% sensitivity, and good internal validity [28].
- Music Performance Anxiety Inventory (K-MPAI): A 40-item questionnaire assessing various aspects of Music Performance Anxiety (MPA), rated on a Likert-type scale with seven options scoring from 0 (strongly disagree) to 6 (strongly agree) [29]. The total score ranges from 0 to 240, with scores above 105 indicating a significant level of MPA [30]. It has high internal validity and correlates with other measures of anxiety [30,31].
- Warwick-Edinburgh Mental Well-Being Scale (WEMWBS): A 14-item questionnaire assessing well-being, rated from 1 (not at all) to 5 (all the time). The total score ranges from 14 to 70, with scores above 51 representing the general population. This scale is known for its good psychometric properties [32].

#### Physiological assessments.

- Blood Pressure (BP): Measured on the left arm while the participant is sitting using a calibrated manual (Prestige Medical) sphygmomanometer with an adult sized cuff.
- Heart Rate (HR) and Oxygen Saturation (SaO2): Measured using a calibrated digital pulse oximeter (Biosync, Model B50DL).
- Respiratory Rate (RR): Measured using BioHarness (Zephyr) for five minutes at rest.
- Peak Expiratory Flow Rate (PEFR): Measured as the best of three attempts while standing, using a Mini-Wright standard range peak flow meter (Clement Clarke).

### Procedure

Each participant was scheduled for three appointments, with a minimum interval of seven days between each visit. Informed consent was obtained during the first visit. At the start of every appointment, participants completed the psychological measures, followed by the physiological assessments. For the intervention group, respiratory rate was also assessed throughout the breathing practice.

Participants in the control group engaged in a 30-minute appointment with the investigator but did not receive any specific coaching or breathing guidance. Those in the intervention group underwent a TBr session facilitated by a certified practitioner, Dr. P. Wheble, conducted in accordance with a standardized protocol (available on request), for each two-hour appointment. Thirty percent of the intervention sessions were moderated by the research supervisor, Dr. C Chapman, to ensure protocol adherence and avoid bias. After data collection, participants completed a 10-minute coaching exercise to set an intention for the breathing session. They completed 45-minutes of active HVB practice, followed by a 15-minute relaxation phase. Standardised music was used in each session (available on request). During relaxation, the practitioner recorded objective observations from the session. Immediately after the session, a brief interview was conducted to document the participant’s experience. The outcome measures were repeated 20-minutes after the intervention for the breathing group.

### Statistical analysis

Data analysis was conducted using the IBM SPSS Statistics for Windows, Version 29.0.2.0 (IBM Corporation, Armonk, New York, USA). Demographic characteristics (Table 1) and baseline measures (Table 2) were compared between groups using Independent Samples Mann-Whitney U tests for continuous variables and Chi-Squared or Fisher’s exact tests for categorical variables. There were no statistically significant differences between the intervention and control groups.

**Table 1:** Demographic characteristics of participants.

Demographics [mean (SD) or count (%)]		Intervention (n = 12)	Control (n = 12)	p-value
Age		33.08 (12.64)	29.58 (5.87)	0.887
Gender	Male	3 (25)	2 (16.7)	1.000
	Female	9 (75)	10 (83.3)	
Caffeine intake (cups/day)		1.83 (1.80)	1.67 (1.23)	0.794
Psychological Therapy	Never	2 (16.7)	3 (25)	0.524
	Previous	6 (50)	8 (66.7)	
	Current	4 (33.3)	1 (8.3)	
History of Panic Attack	Never	7 (58.3)	6 (50)	1.000
	Previous	5 (41.6)	6 (50)	
Time to complete experiment (days)		36.33 (18.51)	27.92 (12.00)	0.200

SD: Standard deviation. Independent Samples Mann-Whitney U Test (continuous data), Chi-square/Fisher’s exact (categorical data).

**Table 2:** Baseline measures of participants.

Measure (score range or units) [mean (SD)]	Intervention (n = 12)	Control (n = 12)	p-value
GAD-7 (0–21)	8.25 (3.70)	8.17 (3.88)	0.799
PHQ-9 (0–27)	7.42 (3.63)	7.42 (4.40)	0.887
SPIN (0–68)	34.33 (9.11)	35.17 (9.32)	0.671
K-MPAI (0–240)	128.83 (32.48)	123.08 (32.75)	0.590
WEMWBS (14–70)	46.58 (5.42)	47.75 (9.39)	0.755
Systolic blood pressure (mmHg)	122.67 (22.94)	112.67 (16.65)	0.266
Diastolic blood pressure (mmHg)	70.83 (10.25)	73.50 (8.27)	0.490
HR (bpm)	73.00 (8.54)	74.17 (16.14)	0.932
Respiratory rate (bpm)	16.45 (2.90)	14.08 (3.21)	0.071
Oxygen Saturations (%)	98.50 (0.67)	97.50 (1.51)	0.078
Peak expiratory flow rate	510.83 (55.51)	504.17 (85.33)	0.713

SD: Standard deviation. Independent Samples Mann-Whitney U (continuous data)

To evaluate the impact of the TBr intervention on each outcome measure over the course of the three sessions, linear mixed models were utilised. The outcome measurements taken at the beginning of each session served as the dependent variables. For any post-hoc pairwise comparisons, a Bonferroni correction was used to adjust for multiple tests and control the overall Type I error rate.

As an exploratory analysis, Friedman’s test was conducted for the TBr group, including both pre and post intervention measures, to test the acute effects of the breathing intervention. There were no post-intervention measures for the control group therefore no group comparisons were made. For any post-hoc pairwise comparisons, a Bonferroni correction was used to adjust for multiple tests and control the overall Type I error rate.

## Results

### Anxiety characteristics

During structured interviews, 15 participants (62.5%) reported the onset of anxiety during adolescence, and 13 (54.2%) experienced anxiety specifically in solo performance settings. The majority, 20 (83.3%), identified the fear of negative criticism as the primary trigger for their anxiety. Preferred coping strategies included planning (7, 29.2%), mindfulness (6, 25%), meditation (4, 16.7%), yoga (3, 12.5%), talking (3,12.5%), and using beta-blockers (1, 4.2%).

### Transformational breath observations

The mean respiratory rate (RR) for the active practice of TBr was 19.39 breaths per minute (SD 2.587). The estimated inhalation/exhalation ratio for TBr ranged from 1.63-1.83. Qualitative data from the intervention group (n = 12) are summarized in Table 3. Participants were observed during the TBr session, and a brief interview was conducted at the end of the session. Experiences were categorized into three main categories: physical symptoms, mental/emotional and spiritual themes. During the interventions, 10 ‘respiratory pauses’ were noted. Respiratory pauses are points in the session where participants breathing rate slowed significantly. The mean RR during these pauses was 8.67 bpm (range 4–12), with the longest pause lasting 120 seconds. These observations indicate continued respiratory activity during ‘pauses.’

**Table 3:** Frequency table of experiences reported during 36 TBr sessions.

Reported Themes or Symptoms		Session 1 (N = 12)	Incidence (%)	Session 2 (N = 12)	Incidence (%)	Session 3 (N = 12)	Incidence (%)
Physical Symptoms	Relaxation	11	91.7%	11	91.7%	12	100.0%
	“Respiratory pause”	1	8.3%	5	41.7%	4	33.3%
	Paraesthesia/Tetany	5	41.7%	2	16.7%	3	25.0%
	Sensation of cold/previous physical trauma	3	25.0%	2	16.7%	0	0.0%
	Dizziness	1	8.3%	0	0.0%	1	8.3%
Mental/ Emotional Themes	Acceptance	9	75.0%	12	100.0%	11	91.7%
	Clarity	8	66.7%	12	100.0%	11	91.7%
	Crying, release or catharsis	6	50.0%	6	50.0%	10	83.3%
	Freedom, joy, love, self-worth.	5	41.7%	9	75.0%	12	100.0%
	Anger, loneliness, shame, grief.	3	25.0%	1	8.3%	3	25.0%
	Worried about doing it right	2	16.7%	0	0.0%	0	0.0%
Spiritual Themes	Connection to others	2	16.7%	3	25.0%	2	16.7%
	Deep awareness of self	10	83.3%	12	100.0%	11	91.7%
	Altered states of consciousness	4	33.3%	6	50.0%	7	58.3%
Adverse Events	Headache	1	8.3%	0	0.0%	0	0.0%

### Effect of a course of three TBr interventions

There were no significant Group or Time main effects, nor Group x Time interactions for any psychological or physiological outcome measures. These findings suggest no improvement in any of the psychological or physiological measures in the breathing group in comparison to the control group.

### Exploratory analysis: Acute effects of TBr

**Fig 1:**
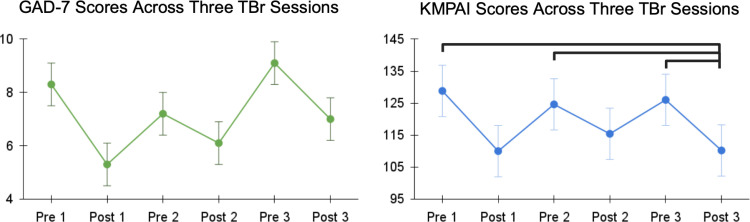
GAD-7 and K-MPAI scores for the TBr group across three sessions, measured before and after each session. Solid line indicates significant difference between time points at p < 0.05, after Bonferroni correction for multiple comparisons.

The pattern of results suggests that music performance anxiety decreased after each TBr session (Fig 1). A significant Time main effect was found for music performance anxiety (K-MPAI) (X2(5) = 20.157, p = .001) in the TBr group. Post hoc Wilcoxon-signed rank tests with Bonferroni correction for multiple comparisons revealed significant differences between K-MPAI scores at time points Post 3 and Pre 3 (z = 3.00, adj. p = .040), Post 3 and Pre 2 (z = 3.27, adj. p = .016) and Post 3 and Pre 1 (z = 2.95, adj. p = .048). Post 3 was the only time point that showed statistically significant reductions in K-MPAI scores when compared with each pre-session score.

A significant Time main effect was found for Generalized Anxiety Disorder (GAD-7) (X2(5) = 12.79, p = .025) in the TBr group. However, no pairwise comparisons survived correction for multiple comparisons, suggesting that there was no acute improvement in generalized anxiety.

## Discussion

This is the first study to explore TBr as a complete HVB practice and to assess the efficacy of TBr as an intervention for anxiety management in PVUs with mild to moderate social anxiety disorder. Contrary to expectations, three sessions of TBr did not yield significant changes in any physiological or psychological measures of anxiety, depression, or well-being in comparison to a waitlist-control group. However, exploratory analyses of changes in psychological measures from before to after each TBr session indicate a significant reduction in music performance anxiety (K-MPAI), as indicated by significantly lower anxiety scores of the Post 3 timepoint, in comparison to Pre 3, Pre 2 and Pre 1. These findings indicate that TBr may produce an acute reduction in music performance anxiety. This pattern may reflect a cumulative effect of TBr or be indicative of participants learning and benefitting from the technique over time. Further research with larger samples would be required to explore this.

The efficacy of a TBr intervention with three sessions spaced one week apart, appears insufficient to achieve a detectable long-term reduction in anxiety measures. Three sessions of TBr were investigated as this is the number of sessions required to learn the technique for independent use [33]. The study was limited in time and resources, so the intervention was capped at three sessions. The gold standard CBT interventions for anxiety disorders typically require 12–15 sessions to achieve a sustained improvement in symptoms [34]. Studies exploring similar HVB interventions for anxiety also required 8–10 sessions [16,17]. This suggests that a longer course of TBr practice may be required to demonstrate lasting psychological improvements.

The qualitative data (Table 3) demonstrate positive effects of TBr on relaxation, acceptance, clarity, self-worth and a deep awareness of self, which appear to increase with practice. The incidence of non-ordinary states of consciousness during the interventions increased in each successive session: 33.3% of participants reported experiencing this in the first session, 50% in the second session and 58.3% in the third session. This may indicate a learned ability to sustain the prescribed breathing pattern as participants gained more competence with the technique. Further exploration and quantification of these experiences is an interesting future avenue of research.

### Strengths and limitations

This study is the first to assess TBr as an intervention for anxiety management in PVUs, using a controlled design with validated psychological and physiological measures. It contributes preliminary data and effect size estimates that can guide future research exploring TBr effects on music performance anxiety. The small sample size (n = 24, with 12 per group) limits the statistical power and generalizability of the findings.

The study design enrolled individuals with mild to moderate symptoms of social anxiety, as indicated by their initial SPIN score. To prevent the enrolment of vulnerable participants, we excluded participants with moderate to severe anxiety or depression as indicated by their initial PHQ-9 and GAD-7 scores. It is possible that the baseline levels of anxiety were not high enough for a significant reduction to be detectable through the measures employed.

The lack of a strictly controlled intervention length, where the TBr group underwent longer sessions (70 minutes including pre and post assessments) compared to 30 minutes for the control group, may have introduced confounding variables. Future research must include a larger sample size and control group design that replicates the experimental conditions and differs only in the intervention that is being evaluated.

Despite these limitations, the study makes a valuable contribution by demonstrating acute psychological changes and providing foundational evidence which warrants further investigation.

## Conclusion

The current study found evidence of acute reductions in performance anxiety following three sessions of TBr. Research of complete HVB practices of this kind is lacking and publication of methodologies and procedures to improve research quality in this field is required. The findings indicate that exploration of conscious breathing interventions for performance anxiety in the pre-performance setting may be of benefit to professional voice users. Further research with a longer intervention period, a larger number of TBr sessions and with individuals exhibiting a broader range of anxiety severity are needed to fully understand the potential benefits of TBr for anxiety management.
